# Factors Associated With Work Engagement of Nurses During the Fifth Wave of the COVID-19 Pandemic in Japan: Web-Based Cross-Sectional Study

**DOI:** 10.2196/45830

**Published:** 2023-11-03

**Authors:** Kei Muroi, Mami Ishitsuka, Tomoko Hachisuka, Itsuka Shibata, Tomohiko Ikeda, Daisuke Hori, Shotaro Doki, Tsukasa Takahashi, Shin-ichiro Sasahara, Ichiyo Matsuzaki

**Affiliations:** 1 Graduate School of Comprehensive Human Sciences University of Tsukuba Tsukuba Japan; 2 Nursing Department Eastern Chiba Medical Center Togane Japan; 3 School of Nursing and Rehabilitation Sciences Showa University Yokohama Japan; 4 Institute of Medicine University of Tsukuba Tsukuba Japan; 5 International Institute for Integrative Sleep Medicine University of Tsukuba Tsukuba Japan

**Keywords:** age, COVID-19, cross-sectional study, engagement, experience, gender, intensive care, Japan, JD-R model, job demands-resources, mental health, nurses, psychiatric, psychological, survey, work engagement

## Abstract

**Background:**

The COVID-19 pandemic has brought to light the prevalence of mental health issues among nurses. Work engagement (WE) is a concept that describes work-related positive psychological states and is of importance within mental health measures. There is, however, a lack of research on factors associated with the WE of nurses during the COVID-19 pandemic.

**Objective:**

We aimed to determine which factors are associated with WE among nurses during the COVID-19 pandemic using the job demands-resources (JD-R) model as a framework.

**Methods:**

A web-based cross-sectional survey was conducted among nurses working in acute care and psychiatric institutions in the prefectures of Chiba and Tokyo in Japan. The survey period occurred between August 8 and September 30, 2021, during a time when the number of patients with a positive COVID-19 infection increased. The 3-item version of the Utrecht Work Engagement Scale (UWES-3) was used to measure WE. Factors such as age, gender, years of experience, affiliated ward, COVID-19–related stress, financial rewards from the government and hospital, encouragement from the government and patients, and workplace social capital were assessed. A total of 187 participants were included in the final analysis. Multiple regression analysis was performed to examine the factors related to WE. Partial regression coefficients (B), 95% CI, and *P* values were calculated.

**Results:**

The mean overall score for the UWES-3 was 3.19 (SD 1.21). Factors negatively associated with UWES-3 were COVID-19–related stress on work motivation and escape behavior (Β –0.16, 95% CI –0.24 to –0.090; *P*<.001), and factors positively associated with UWES-3 were affiliation of intensive care units (Β 0.76, 95% CI 0.020-1.50; *P*=.045) and financial rewards from the government and hospital (Β 0.40, 95% CI 0.040-0.76; *P*=.03).

**Conclusions:**

This study examined factors related to WE among nurses during the COVID-19 pandemic using the JD-R model. When compared with findings from previous studies, our results suggest that nurses’ WE was lower than before the COVID-19 pandemic. Negative motivation and escape behaviors related to COVID-19 were negatively associated with WE, while there were positive associations with financial rewards from the government and hospital and affiliation with an intensive care unit. Further research into larger populations is needed to confirm these findings.

## Introduction

### Mental Health of Nurses in the COVID-19 Pandemic

The COVID-19 pandemic has exacerbated the mental health of health care professionals [1-3]. Despite the implementation of measures to limit the spread of the infection, such as physical distancing, mask-wearing, and vaccination, the conclusion of the pandemic remains uncertain [4]. Health care professionals are at an elevated risk of mental health issues not only during the pandemic but also in its aftermath, underscoring the necessity of psychological support for health care workers at such demanding times [5]. Therefore, it is important to implement mental health support measures for health care workers during and after the COVID-19 pandemic. Among the various health care professions, nurses are the most populous, have the most patient contact, and spend the most time involved in patient care. The negative impact of the pandemic on the mental health of nurses is of particular concern, as a decrease in the number of nurses in medical institutions can lead to an increase in medical errors and a decline in the quality of medical care [6,7]. High levels of stress and anxiety among health care workers are associated with lower morale, lower job satisfaction, and absenteeism [8]. Therefore, maintaining the mental health of nurses is essential not only for the well-being of the nurses themselves but also for the health and safety of patients, the functioning of the health care system, and the financial health of health care institutions.

### Work Engagement

Despite the pressing need for psychological intervention for nurses, who have been particularly affected by mental health problems during the COVID-19 pandemic, there is a lack of evidence for such interventions. Positive psychology, which focuses on human strength and optimal functioning, has been suggested as a potential mental health measure [9]. Work engagement (WE), which is a psychological state comprising vigor, absorption, and dedication, has been proposed as an opposite concept to burnout from the perspective of positive psychology in the workplace [10]. Workers with high WE have less psychological distress and lower rates of absenteeism and intention to leave [11,12]. The job demands-resources (JD-R) model, which posits that WE is determined by job demands and personal and workplace resources, has been shown to be valid when investigating the WE of nursing populations [13-15].

The JD-R model framework probes the relationship between job characteristics and workers’ psychological states. The JD-R model proposes 2 main pathways, linking job resources and personal resources to motivational outcomes such as WE and high job demands to psychological strain. Job demands are defined as the physical, psychological, social, or organizational aspects of a job that require continuous physical and psychological effort. Job resources refer to the physical, psychological, social, or organizational aspects of work that function in achieving goals, reducing work demands, and stimulating personal growth, learning, and development. According to a review by Bakker and Demerouti [13], job demands pertain to the quantitative and mental workload of the job, while job resources include access to resources, compensation, and support from colleagues. When job demands and control are not balanced, WE is reduced because employees feel stressed and are unable to use their resources. An important aspect of the JD-R model is that it posits that only ample job resources and not low job demands positively impact WE [16]. Interventions focusing on WE based on the JD-R model may be useful in promoting the mental health of nurses; however, there is a lack of research examining relevant factors for WE among nurses, especially during the COVID-19 pandemic.

In Japan, where the COVID-19 pandemic is currently ongoing, nurses’ mental health is reported to be deteriorating [2]. Health care workers, including nurses, who are involved in COVID-19 care may experience psychological burdens, such as fear of infection and feelings of isolation [2,17,18]. However, it is unclear whether these factors are associated with WE in this population. In addition, the government implemented the “support bonus for novel coronavirus response workers” in 2021 [19], which provides special remuneration to health care workers involved in the care of patients with COVID-19. Social networking campaigns were also conducted to convey messages of gratitude to health care workers involved in medical treatment [20]. Private companies and local governments also expressed appreciation for health care workers. While financial rewards and appreciation within the workplace are associated with WE [21-23], there is a lack of research on the relationship between these factors and WE among nurses during the COVID-19 pandemic.

It is important to identify the specific factors that are associated with nurses’ WE during the COVID-19 pandemic in order to maintain and improve their mental health for this and possible future pandemics. Therefore, we aimed to determine which factors were associated with WE among nurses during the COVID-19 pandemic, using the JD-R model as a framework.

## Methods

### Study Design

**Figure 1:**
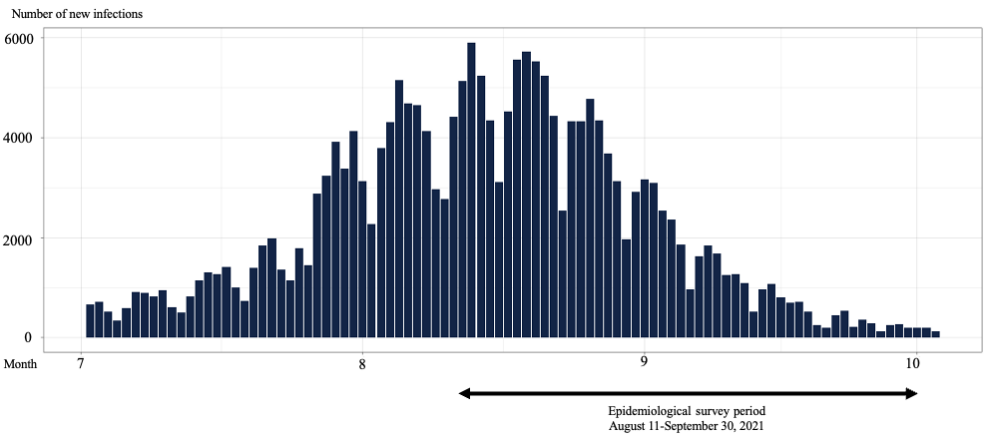
Number of newly confirmed COVID-19 cases in Tokyo in 2021.

**Figure 2:**
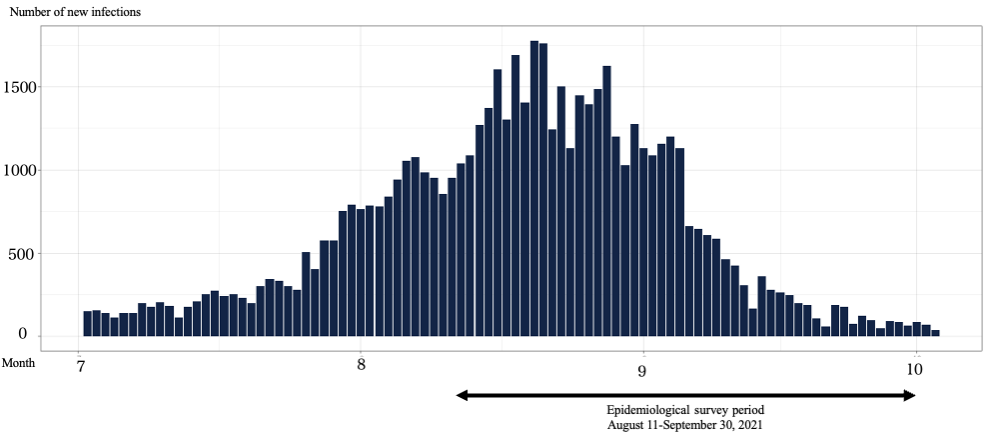
Number of newly confirmed COVID-19 cases in Chiba in 2021.

A cross-sectional survey using Google Form (Google LLC) was conducted in a target population of 400 nurses working in acute care and psychiatric institutions in Chiba and Tokyo prefectures in Japan. At each hospital, the principal investigators (study coauthors) methodically disseminated details about the research and encouraged participation in the study. The workforce of employed nurses in Japan totals around 1.2 million individuals [24]. Given this population size and using a significance level of 5%, the necessary sample size was calculated to be 385 individuals; thus, the number of participants was established at 400. The research participants included only individuals from the nursing profession. Participants with incomplete items in the instruments were excluded from the analysis. Nurses were recruited for this study through posters and were offered a reward of a gift card (worth ¥500 [US $3.31]) for their participation. The survey was conducted from August 8 to September 30, 2021, during the “fifth wave” of the COVID-19 pandemic, when the number of positive cases was increasing [25,26]. During this time, the highly infectious Delta variant of the SARS-CoV-2 virus replaced earlier strains, leading to an unprecedented rapid and widespread infection in many areas of Japan. Both Chiba and Tokyo prefectures, which have high population densities, had strained health care systems due to high numbers of infections. Graphs illustrating the incidence of new COVID-19 cases and the temporal span of the study are presented using open-access data graciously provided by the Ministry of Health, Labor, and Welfare (Figures 1 and 2) [27].

### Measurements

#### Personal Resources

Every questionnaire, serving as a research instrument, was written and responded to exclusively in the Japanese language. Gender was divided into 3 categories: male, female, and other. In Japan, individuals are eligible to pursue a career in nursing upon attaining a minimum age of 20 years. Regarding the classification of age groups, we adhered to the widely accepted 10-year age brackets typically used in nursing surveys [28-30]. Therefore, the designated age categories encompassed 20-29, 30-39, 40-49, 50-59, and 60 years and older.

#### Occupational Attributes

Affiliation was divided into 5 categories: general ward, intensive care unit (ICU), COVID-19 unit, psychiatric hospital, outpatient, home visit, and hemodialysis unit. Years of nursing experience were divided into 4 categories: less than 1 year, 1-5 years, 5-10 years, and more than 10 years.

#### WE Measure

The 3-item version of the Utrecht Work Engagement Scale (UWES-3) [31] was used to assess WE. This questionnaire assesses an individual’s active engagement and energy at work through 3 subscales: vigor, dedication, and absorption. Respondents rated their vigor, dedication, and absorption on a 7-point scale ranging from “never” (0) to “always” (6). The total score was calculated by dividing the sum of the subitems by 3, with a possible range of 0-6. The UWES-3 has fewer items than the 17-item version of UWES and the 9-item version of UWES and was chosen to reduce respondent burden [32]. The Cronbach α for the UWES-3 in this study was .882.

Job demands were measured using the COVID-19-related stress questionnaire developed by Awano and colleagues [2]. This questionnaire consists of 3 subscales: anxiety and fear of infection and death (10 items), isolation and unreasonable treatment (5 items), and motivation and escape behavior at work (3 items). Respondents rated each subscale on a 4-point scale ranging stress related to COVID-19 as a job demand from “none” (0) to “true” (3), with total scores ranging from 0 to 30 for anxiety and fear of infection or death, 0 to 15 for isolation and unreasonable treatment, and 0 to 9 for work motivation and escape behaviors. The Cronbach α for the questionnaire in this study was .902.

#### Workplace Environment

Workplace social capital (WSC) is a concept that includes characteristics of specific social groups, such as workplace relationships, social relations and networks, norms of reciprocity, and trust. The Japanese version of the WSC scale, created by Kouvonen and colleagues [33], was used for measuring WSC [34]. Responses are made using a 4-point scale ranging from “I don’t think so” (1) to “I agree” (4), and the average score is calculated. Scores range from 1 to 4 points. The Cronbach α for the WSC in this study was .926.

### Statistical Analysis

**Figure 3:**
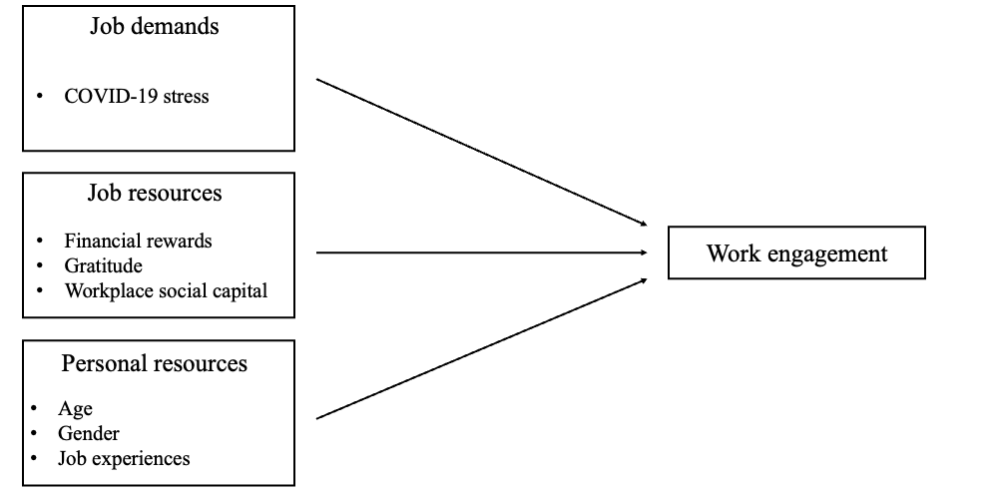
Factors related to work engagement based on the job demands-resources model.

Multiple regression analysis was conducted with the UWES-3 as the dependent variable to identify factors associated with WE. Independent variables included basic attributes, occupational attributes, job demand, and job resources (Figure 3). UWES-3, COVID-19–related stress, and WSC were incorporated into the multilinear regression as continuous variables, whereas age (reference: 20-29 years), gender (reference: female), years of job experiences (reference: <1 year), ward affiliation (reference: general ward), gratitude from the government, private sector, and patients (reference: no), and reward from hospitals or the government (reference: no) were treated as categorical variables. Partial regression coefficients (B), 95% CIs, and *P* values were calculated, and adjusted *R*^2^ was calculated as the goodness of fit of the model. All analyses were conducted at a significance level of .05 and using R software (version 4.0.3; R Foundation for Statistical Computing).

### Ethical Considerations

The web-based survey clearly stated that participation was voluntary and anonymous and that respondents’ privacy would be respected and data strictly controlled. The research proposal was reviewed and approved by the Ethics Committee of the University of Tsukuba (approval 1642), and all procedures were conducted in accordance with the ethical standards of national research committees and the Declaration of Helsinki.

## Results

### Demographics

A total of 209 nurses participated in the study. Of these, 22 participants with missing survey items were excluded, resulting in a final sample of 187 (165 women and 22 men; a valid response rate of 46.8%, 187/400). Table 1 shows the demographic characteristics of the study population. Table 2 shows the UWES-3 results. The mean of the overall score for UWES-3 was 3.19 (SD 1.21), and the mean scores of the subitems were 3.06 (SD 1.38) for absorption, 3.72 (SD 1.29) for dedication, and 2.79 (SD 1.37) for vigor.

**Table 1:** Participant demographic data (n=187).

Characteristic		Value
**Age, n (%)**		
	20-29 years	54 (28.9)
	30-39 years	32 (17.1)
	40-49 years	57 (30.5)
	50-59 years	33 (17.6)
	>60 years	11 (5.9)
Gender (female), n (%)		165 (88.2)
**Job experiences, n (%)**		
	<1 years	9 (4.8)
	1-5 years	39 (20.9)
	5-10 years	42 (22.5)
	>10 years	97 (51.9)
**Affiliations, n (%)**		
	General ward	44 (23.5)
	ICU^a^	11 (5.9)
	COVID-19 unit	28 (15)
	Outpatient and home-visit nursing care and hemodialysis unit	73 (39)
	Psychiatric hospital	31 (16.6)
Gratitude from the government, private sector, and patients (yes), n (%)		148 (79.1)
Reward from hospitals or the government (yes), n (%)		123 (65.8)
**COVID-19-related stress**		
	Anxiety and fear of infection and death, mean (SD)	19.5 (8.49)
	Isolation and unreasonable treatment, mean (SD)	1.73 (2.43)
	Motivation and escape behavior at work, mean (SD)	3.48 (2.60)
Workplace social capital, mean (SD)		3.47 (0.79)

^a^ICU: intensive care unit.

**Table 2:** The result of the 3-item version of the Utrecht Work Engagement Scale (UWES-3; n=187).

UWES-3	Value, mean (SD)
Overall	3.19 (1.21)
Dedication	3.06 (1.38)
Absorption	3.72 (1.29)
Vigor	2.79 (1.37)

### Multiple Regression Analysis

Table 3 shows the results of the multiple regression analysis. The factors negatively associated with UWES-3 were motivation and escape behavior at work (Β –0.16, 95% CI –0.24 to –0.090; *P*<.001), and factors positively associated with UWES-3 were affiliation with ICUs (Β 0.76, 95% CI 0.020-1.50; *P*=.045) and financial rewards from the government and hospital (Β 0.40, 95% CI 0.040-0.76; *P*=.03). Gratitude from the government, private companies, and patients was not statistically associated with UWES-3 (Β 0.060, 95% CI –0.35 to 0.48; *P*=.77). The adjusted *R*^2^ value was 0.235.

**Table 3:** Multiple regression model for the 3-item version of the Utrecht Work Engagement Scale (UWES-3).

Independent variable			UWES-3		
			Β (95% CI)		*P* value
**Age (reference: 20-29 years)**					
	30-39 years	0.22 (–0.37 to 0.81)		.46	
	30-39 years	0.22 (–0.37 to 0.81)		.46	
	40-40 years	–0.050 (–0.66 to 0.57)		.88	
	50-59 years	0.16 (–0.49 to 0.80)		.63	
	>60 years	0.33 (–0.55 to 1.21)		.46	
**Gender (reference: female)**					
	Male	–0.52 (–1.05 to 0.01)		.05	
**Job experiences (reference: <1 year)**					
	1-5 years	–0.82 (–1.64 to 0.00)		.05	
	5-10 years	–0.80 (–1.69 to 0.09)		.08	
	>10 years	–0.23 (–1.19 to 0.72)		.63	
**Affiliations (reference: general ward)**					
	ICU^a^	0.76 (0.020 to 1.50)		.045	
	COVID-19 unit	0.22 (–0.36 to 0.80)		.46	
	Outpatient and home-visit nursing care and hemodialysis unit	–0.25 (–0.73 to 0.22)		.29	
	Psychiatric hospital	0.00 (–0.56 to 0.55)		.99	
Gratitude from the government, private sector, and patients (reference: no)			0.060 (–0.35 to 0.48)		.77
Reward from hospitals or the government (reference: no)			0.40 (0.04 to 0.76)		.03
**COVID-19–related stress**					
	Anxiety and fear of infection and death	0.010 (–0.01 to 0.03)		.38	
	Isolation and unreasonable treatment	0.050 (–0.02 to 0.12)		.16	
	Motivation and escape behavior at work	–0.16 (–0.24 to –0.09)		<.001	
Workplace social capital			0.17 (–0.06 to 0.41)		.15

^a^ICU: intensive care unit.

## Discussion

### Overview

In this study, we examined the WE of nurses during the COVID-19 pandemic using the JD-R model. According to a 2019 survey conducted by the Japan Institute for Labor Policy and Training (JILPT), the average WE of health care professionals in Japan was 3.51 for absorption, 2.84 for vigor, 4.03 for dedication, and 3.46 overall [35]. Compared to the JILPT study, which was conducted before the COVID-19 pandemic, the WE measured in this study was lower overall and in the subitems. It should be noted that the previous study used the longer 17-item version of UWES, so while our findings suggest that WE was lower during the COVID-19 pandemic, the results of this study (which used the UWES-3) and the previous one are not directly comparable. In a cross-sectional study from Spain that used the UWES-3 to measure nurses’ WE during the COVID-19 pandemic, the overall UWES-3 mean was approximately 2.44 [36], which was also lower than in another study conducted before the COVID-19 pandemic (using the 9-item version of UWES; the overall mean was 4.001) [37]. These results also suggest that WE among nurses may also have decreased during the COVID-19 pandemic in countries outside of Japan.

During the COVID-19 pandemic, WE tended to decrease with stronger negative motivation and escape motivation. There is limited previous research on the relationship between work motivation and WE, and to the best of our knowledge, only 2 studies involving nurses have been reported [38,39]. We are the first to show that COVID-19–related negative motivation and escape behavior are associated with decreased WE. Workplace infection control measures may be important in decreasing WE during the COVID-19 pandemic, as reported by nurses who have witnessed their colleagues contracting COVID-19 due to occupational exposure [40]. Affiliation with an ICU was positively associated with WE. Nurses’ professional factors may be associated with WE, as many nurses in ICUs are dedicated to the care of critically ill patients. State and hospital rewards were also positively associated with WE; previous studies before and after the COVID-19 pandemic have also reported that financial rewards for nurses are associated with work motivation [41,42], which is consistent with our results. Nurses who worked during the COVID-19 pandemic may perceive financial rewards as an important resource. In their study of Japanese health care workers during the COVID-19 pandemic, Morishita and colleagues [43] found that many health care workers considered financial rewards important in performing their jobs. Our results showing that financial rewards are associated with nurses’ WE confirm those of Morishita and colleagues [43]. On the other hand, appreciation from the government, the private sector, and patients was not associated with WE. A possible psychological mechanism for improving WE through appreciation is that workers who receive appreciation may perceive that the interaction between the work environment and themselves is successful, which promotes feelings of competency and ability in their job, ultimately increasing WE [44]. However, previous studies have found that appreciation among colleagues within the workplace, rather than appreciation from external sources such as the government, private sector, and patients, is associated with WE. Thus, acts of appreciation within the workplace and among colleagues may be necessary to increase WE among health care professionals.

### Implication

This study provides useful insights into the WE-related factors among nurses during the COVID-19 pandemic and has implications for how nurses’ WE might be improved in times of such a profound medical crisis. While cognitive behavioral therapy (CBT) has been shown to enhance nurses’ WE with the goal of augmenting personal resources [45], the effect sizes in CBT intervention studies have been small, and it may be difficult to implement CBT for all nurses involved in COVID-19 care. Therefore, other realistic methods for increasing WE among nurses are needed to help support their mental well-being. The assurance of adequate infection control measures in hospital wards may contribute to nurses’ motivation to work and increase WE. In a study conducted during an influenza pandemic, confidence in safety, risk perception, previous training, general and role knowledge, and confidence in skills were identified as facilitators of nurses’ work motivation. Thus, during pandemic times, adequate infection control measures in hospital wards, training in infection control, and risk awareness may be important for maintaining work motivation [46]. Financial rewards are also important for improving nurses’ WE. It remains important for the government to provide financial support to nurses working with patients with COVID-19.

### Limitations

There are several limitations to this study. First, it is a cross-sectional study, so causal relationships cannot be determined. Additionally, the response rate was 46.8% (187/400), which may indicate a selection bias and necessitate improvement in the collection method. It is also possible that the participants in this study are not representative of hospitals nationwide. There is a possibility of recall bias as well, as only subjective evaluations of rewards and appreciation were made. Finally, the model goodness of fit for the multiple regression analysis was not ideal, with an adjusted *R*^2^ of 0.235. It is, therefore, important to explore further factors associated with WE in larger populations in the future.

### Conclusion

In this study, we examined factors related to WE among nurses during the COVID-19 pandemic using the JD-R model. The results suggest that nurses’ WE is likely to be lower than before the COVID-19 pandemic. Negative motivation and escape behaviors related to COVID-19 were negatively associated with WE, while financial rewards from the government and hospital and affiliation with an ICU were positively associated with WE. Further research using larger study populations is needed to confirm these findings; however, they offer useful insights into how we can support the mental health of nurses in difficult times.

## Abbreviations

CBT: cognitive behavioral therapy
ICU: intensive care unit
JD-R: job demands-resources
JILPT: Japan Institute for Labor Policy and Training
UWES-3: 3-item version of the Utrecht Work Engagement Scale
WE: work engagement
WSC: workplace social capital

## Data Availability

The data sets generated during and analyzed during this study are available in the Zenodo repository [47].
